# Process Optimization, Characterization and Antioxidant Capacity of Oat (*Avena Sativa* L.) Bran Oil Extracted by Subcritical Butane Extraction

**DOI:** 10.3390/molecules23071546

**Published:** 2018-06-27

**Authors:** Xiao Guan, Shengye Jin, Sen Li, Kai Huang, Jing Liu

**Affiliations:** 1 School of Medical Instruments and Food Engineering, University of Shanghai for Science and Technology, Shanghai 200093, China; Dumpking@163.com (S.J.); lisen_1027@126.com (S.L.); hkjn1990@163.com (K.H.); 2 College of Information Engineering, Shanghai Maritime University, Shanghai 200135, China; jingliu@shmtu.edu.cn

**Keywords:** oat bran oil, subcritical butane extraction, optimization, antioxidant capacity

## Abstract

Oat bran is a traditional agricultural byproduct and rarely used in edible oil processing. In this paper, oat bran oil (OBO) was firstly extracted by subcritical butane extraction (SBE) and the extraction process was optimized using response surface methodology. Three variables involving liquid-to-solid ratio, extraction time and extraction temperature were studied. The optimum conditions for extraction of OBO were obtained as follows: liquid-to-solid ratio 4.30, extraction time 48.15 min, and extraction temperature 46.52 °C. Based on this, an alternative method (SBE-e) for cosolvent (ethanol) was proposed to improve SBE method. Compared to conventional hexane extraction (CHE), the SBE-e had significant effect on yield, bioactive compounds (phytosterols and phenols) and antioxidant capacity (AC) in the extracted OBO. The results indicated that the proposed methods were appropriate for OBO extraction. Additionally, OBO had the potential to be an acceptable substitute for edible oil, owing to its desirable physicochemical characteristics, a balanced fatty acids composition and high antioxidant capacity.

## 1. Introduction

Oat (*Avena sativa* L.) is a popular cereal grain of *Poaceae* (*Graminaceae*) family, with a planting area of nearly 10 million hectares globally. Compared with the other cereal grains, oats have high amounts of oil in the endosperm. Oats have a variety of uses in functional food and daily life. Many forms of oat are processed into products including oat germ, oat kernel and oat starch. Some personal daily care products such as shampoos, conditioners, shaving gels and moisturizers contain oats [1].

Oat bran is the outer layer of the oat kernel and is mainly a processed byproduct used as poultry feed in the past. Recently, researchers have found that the bran contains 13–20 g/100 g protein, 2–12 g/100 g fat, 60 g/100 g carbohydrates, 11 g/100 g dietary fiber and 10.4 g/100 g β-glucan [2]. The most important and enthusiastic topic is dietary fiber including both soluble and insoluble dietary fiber [3]. Based on various scientific research, the oat-soluble fiber has great effect on reducing the risk of cardiovascular disease, obesity, and diabetes. Oat contains high levels of fat, mainly derived from oat bran [4], which makes it possible to be a functional food product. However, oat bran has not yet been developed for a source of edible oil. Oat oil has been proved to lower the plasma and liver cholesterol concentrations in hypercholesterolemic rats [5]. Therefore, this study is focused on the extraction and properties of oat bran oil (OBO). It is significant for enhancing the utilization of oat bran, which could expand the demand for oat products.

Conventional oil extraction technologies include mechanical pressing, chemical extraction, and supercritical CO_2_ extraction. Mechanical pressing method consumes too much energy with a low extraction yield. Chemical extraction involves some organic solvent such as hexane, which is used widely in the production of oils. However, the insufficient separation process causes residual solvents and is harmful to the environment and human health to some degree. Supercritical CO_2_ extraction is a representative supercritical fluid extraction and the product obtained is nontoxic with zero residual organic solvent [6], while high pressure and long extraction time are required.

Subcritical fluid extraction is an alternative method for the extraction and separation of functional components. Research has mainly focused on flavors [7], special natural compositions [8] and functional edible oils [9]. Butane is one of the most commonly used subcritical fluids in the extraction of oils, owing to a high solvation power under lower critical pressures and temperatures [10]. Compared to other techniques, subcritical butane extraction (SBE) has safe, efficient, and environmental compatibility. The extraction is a continuous counter current process in which the solvent can be removed completely by system depressurization [11]. In addition, butane has the advantages of cheap price and no residue in the products for its low boiling point.

Subcritical butane extraction (SBE) has been applied to the extraction of edible oil in recent years, but no reports have been published about the OBO produced by SBE. Thus, the primary work of the study is aimed at optimizing the SBE process of OBO using response surface methodology (RSM) and make tentative improvement on this basis. 

Traditionally, edible oil is produced by extraction with a single solvent after the material has been pretreated simply. Cosolvents (or solvent modifiers) would increase the efficiency of extraction to a certain extent [12]. It has been reported that the addition of ethanol to supercritical CO_2_ extraction enhanced the antioxidant capacity with the increase of total phenolic content in oil [13]. Ethanol has been successfully employed in the extraction of rice bran oil instead of conventional extraction using hexane. 

For this reason, the objective of the investigation is to optimize the SBE process of OBO and try the promising alternative method with the addition of ethanol based on SBE. OBO extracted by these two methods were compared with that of conventional hexane extraction (CHE) on characterization and antioxidant capacity, to develop an efficient and biological approach for OBO.

## 2. Results and Discussion

### 2.1. Response Surface Optimization of SBE

#### 2.1.1. ANOVA Analysis and the Model Fitting

The range and center point values of three independent variables was determined based on the single-factor experiments, including (*X*_1_) liquid-to-solid ratio, (*X*_2_) time and (*X*_3_) temperature. Table 1 details the BBD matrix and response values carried out for developing the model, consisting 17 experiment points carried out in random order. The extraction yield was taken as the response variable in the optimization experiments.

The second-order regression equation for extraction of OBO was obtained based on the experimental data with the multiple regression analysis. It is provided below as Equation (1):

*Y* = 5.92 + 0.15*X*_1_ + 0.28*X*_2_ + 0.42*X*_3_ + 0.045*X*_1_*X*_2_ − 0.16*X*_1_*X*_3_ − 0.075*X*_2_*X*_3_ − 0.19*X*_1_^2^ − 0.33*X*_2_^2^ − 0.53*X*_3_^2^ (1)

The analysis of variance results for the model are shown in Table 2. The coefficient of determination (*R*^2^) of the model was calculated and come to 0.9590, indicating that the model could explain the relationship among the independent variables. The model *F*-value of 18.18 and *p*-value of 0.0005 demonstrated the model was statistically significant. Additionally, the lack of fit was not significant (*p* > 0.05), suggesting the equation was adequate for determining the effects of the independent variables on the extraction yield of OBO.

The ANOVA of the linear, quadratic and interaction terms of the model are shown in Table 2 as well. The significance of each regression coefficient was evaluated using *F*-value and *p*-value. Specifically, two linear (*X*_2_ and *X*_3_) and two quadratics (*X*_2_^2^ and *X*_3_^2^) were highly significant model terms (*p* < 0.01). The linear *X*_1_ and quadratic *X*_1_^2^ were significant model terms (*p* < 0.05). Three interaction parameters (*X*_1_*X*_2_, *X*_1_*X*_3_ and *X*_2_*X*_3_) were insignificant terms (*p* > 0.05).

#### 2.1.2. Response Surface Analysis

**Figure 1:**
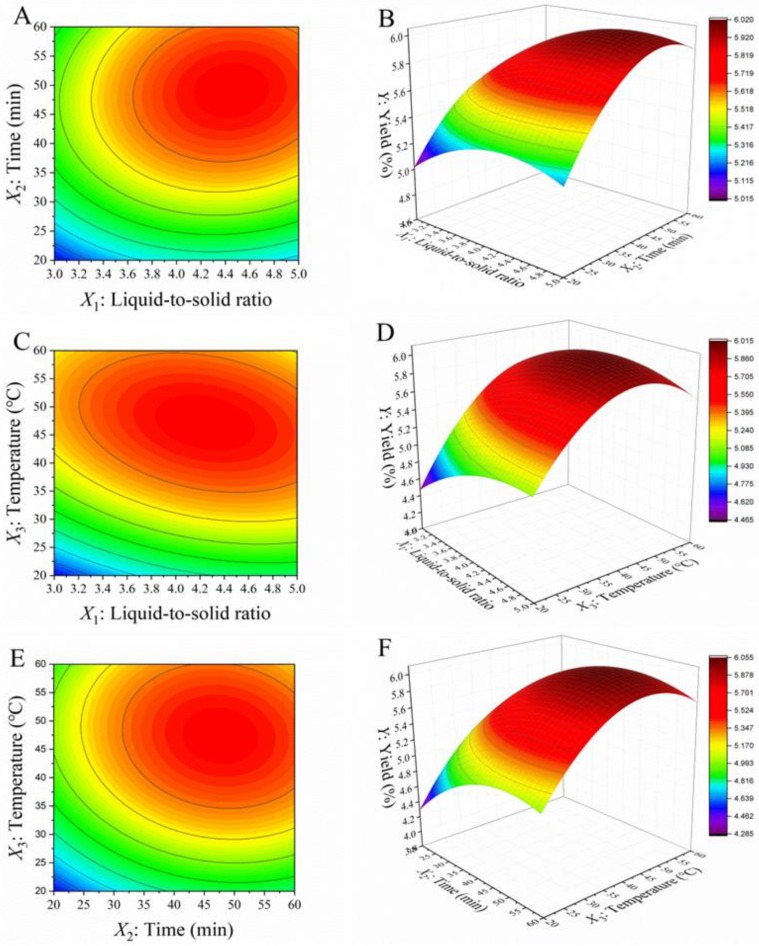
Contour plots (**A**,**C**,**E**) and response surface plots (**B**,**D**,**F**) of the extraction yield affected by liquid-to-solid ratio, time, and temperature.

The three-dimensional (3D) response surface and two-dimensional (2D) contour plots were constructed based on the regression equation. As shown in Figure 1, two variables within the experimental range were described in surface plots while holding the third variable at zero level. The effects of liquid-to-solid ratio and extraction time on the extraction yield at a certain extraction temperature (40 °C) were shown in Figure 1A,B. The yield increased with increasing liquid-to-solid ratio and extraction time, whereas the extraction time is worthier of attention relatively. Oil yield reached to a plateau at approximately >45 min with the increase of liquid-to-solid ratio. The result implied that the system reached saturation with enough extraction time. This effect can be attributed to the fact that solvents penetrate the oil cellular structure at a rapid rate, which stabilizes gradually with increased time. In this case, the highest yield was obtained with a liquid-to-solid ratio and an extraction time of approximately 4.45 and 49.1 min. It was similar to the report for the subcritical fluid extraction of seed oil from *Nitraria tangutorum* [14].

Figure 1C,D shows the contour plot and the response surface plot based on liquid-to-solid ratio and extraction temperature at a constant extraction time (40 min). As discussed above, the extraction temperature had high significant effect (*p* < 0.01) on the oil yield. An increase in temperature leads to an increase in the oil solubility of the solvent, resulting in an increased oil diffusion coefficient. Meanwhile, the oil yield increased with the increase of solvent, due to the diluted solution provided a higher driving force and lower viscosity for extraction. The interaction between liquid-to-solid ratio and temperature obtained in this study was consistent with the report by Mello et al. [15] indicating that the solubility of solvent to oil is the result of the amount and high mass transfer temperature.

The effects of extraction time and temperature on the extraction yield at a constant liquid-to-solid ratio (4:1) were shown in Figure 1E,F. It can be concluded that prolonged extraction time has no effect on improving the extraction efficiency, the yield increased with increasing extraction time especially from 20 to 40 min but slowly from 40 to 60 min. Thus, the most efficient extraction period for OBO under specific conditions was about 40–45 min. This observation was attributed to the fact that the extraction of oil from oat bran mainly appeared in the solvent penetration. Similar investigation reported the extraction is more effective in the first 30 min for flaxseeds oil [16].

#### 2.1.3. Optimization of Extraction Conditions

The optimal conditions for extraction yield obtained by the Design Expert software was as follows: a liquid-to-solid ratio of 4.30:1, an extraction time of 48.15 min, and an extraction temperature of 46.52 °C. Under these conditions, the predicted extraction yield was 6.07%. The optimal condition was slightly modified to a liquid-to-solid ratio of 4.3:1, an extraction time of 48 min, and an extraction temperature of 47 °C, for convenience purposes. The results confirmed that the actual experimental value (6.05%, *n* = 3) was consistent with the predict value (6.07%). It confirmed that the response model was reliable for the analysis and prediction of OBO extraction.

### 2.2. Comparisons on Oil Yield and Fatty Acid Composition

As discussed above in this research, three methods for the extraction of OBO were used as described in the methods section. Specifically, the subcritical butane extraction under optimal conditions was recorded as SBE in short. Based on it, ethanol (5% mixed solvent) was introduced in SBE to evaluate the efficiency of the extraction with the assistance of cosolvents, marked as SBE-e. Conventional hexane extraction (CHE) was employed as a contrast in this passage.

Table 3 shows the extraction yield and fatty acid composition of the oils obtained by these three methods. The SBE method produced a 6.05% yield and it was higher than that of the CHE (5.75%) method. It suggested that the subcritical fluid such as butane has a lower viscosity and higher diffusion coefficient in oil than conventional organic solvents, which was similar to the research on *N. tangutorum* seeds oil extraction [14]. The addition of ethanol to butane resulted in a higher yield (6.42%) compared to the CHE method, with the amount of oil increased by almost 12%. This can be explained as the fact that small amounts of ethanol (5%) have the capacity to provide an increase in the yield at a suitable pressure and temperature condition, due to the ability of the solvent in forming hydrogen bonds as an aprotic solvent [17]. Additionally, the addition of ethanol reduces the amount of butane used and the price of pure butane is relatively expensive, which increases the economic benefit to a certain extent. Therefore, the SBE-e method seems to have more advantages in the aspects of oil yield, energy consumption, economy, and environmental protection.

Major fatty acids composition and content of the oils obtained by these three methods were also shown in Table 3. The results showed that all these oils were rich in oleic acid (C18:1) (43.53–44.13%) and linoleic acid (C18:2) (32.54–33.22%), making up the main components of unsaturated fatty acid in OBOs. It has been reported that oleic acid plays an important role in the prevention of cardiovascular disease and improving stability for cooking [18]. Linoleic acid is another main fatty acid and it is an important polyunsaturated fatty acid (PUFA), also known as omega-6 fatty acid. It helps to the growth of bone and hair, keep stable metabolism, and regulate the reproductive system [19]. The appropriate proportion between oleic acid and linoleic acid makes OBO a special component for functional applications as edible oil.

It can be concluded that the fatty acid contents of OBOs obtained by three different methods were similar in general, while there were some slight differences in oleic acid and linoleic acid for the oils obtained by the SBE-e method. This could be attributed to the high polarity of ethanol as alcoholic solvents, but it has no effect on the fatty acid composition. Other researchers have found that the fatty acids were non-selectively extracted and isomerization or oxidation of fatty acids did not occur under the extraction conditions.

### 2.3. Physicochemical Characteristics of the Oils

The physicochemical characteristics of OBO extracted by three different methods at the optimal conditions are shown in Table 4. The acid value and peroxide value are widely used for oil quality determinations, measuring the amount of free fatty acids present and the amount of primary oxidation in the oil respectively. These values were susceptible to change with the addition of storage time. Thus, all the oil samples obtained from different extraction methods were determined immediately. As Table 4 shows, the acid value, peroxide value, and iodine value of the OBO by SBE method were 7.16 mg/g, 0.74 meq. O_2_/kg of oil and 102.78 g/100 g of oil, similar to the values of the other two methods. It also indicated that there were no significant differences on these three parameters (*p* > 0.05) and these may be related to the quality of materials used in extraction not the extraction process. The high acid value can be attributed to the higher lipase activity of oat bran, which hydrolyzed triglyceride into free fatty acids before experiments. Sufficient enzyme passivation and an appropriate refining process can make the acid value reach the allowable limits [20]. The low peroxide value (0.68–0.81 meq. O_2_/kg oil) can be explained to the assistance of antioxidative substance such as phytosterols, tocopherol and phenols content. In addition, the oxidization could be further avoided during subcritical fluid extraction. The iodine value of the OBO was 102.78–103.63 g/100 g of oil and it mainly because of its high content of UFA, which implied OBO have a good stability for storage and processing.

Oryzanol and squalene are two bioactive compounds, mainly enriched into rice bran oil and shark liver oil. The results showed that the OBO had lower levels of oryzanol (4.16–4.40 mg/g) and a small amount of squalene (43.40–43.53 mg/kg). This phenomenon can be explained by the fact that rice bran is one of the richest sources of oryzanol; in addition, isolation of oryzanol has been done by preparative HPLC and most of them (about 90%) can be recovered [21]. The squalene content in OBO was consistent with the report by Cayula and García [22], squalene was in relatively high quantities (0.2–7.5 g/kg) within the olive oil but only 0.02–0.3 g/kg in other vegetable oils. Table 4 also lists the contents of other nutraceutical compounds (phytosterols, tocopherols and total phenols) in the samples obtained by three different methods. It was worth noting that the oil obtained by the SBE-e method has significant difference (*p* < 0.05) in the content of phytosterols, tocopherols and total phenols. As can be seen, OBO was not rich in tocopherols (15.77–18.69 mg/100 g) and the content was lower than the results of the previous report [5]. The main reason is probably that the source of the oat bran was from different varieties of species, with the different conditions of climate, storage, and processing. The phytosterols and total phenols content of SBE-e OBO were 2.99 mg/g and 9.63 GA mg/100 g, which was higher than that of other methods. These results indicated that the SBE method had no significant effect on the content of phytosterols and phenols in the extracted oil, but the oil samples had a higher content of that with the cosolvent of ethanol. It had a same tendency as much previous research. In previous studies, it has been reported that the levels of minor compounds in the extracted oil increased when ethanol or isopropanol was used [23]. This effect can be attributed to two aspects: the first one is ethanol is a typical short-chain alcohol, having the high polarity to extract higher amounts of nutritional material into the extracted oils; the second one is the higher extract yields of SBE-e than other two methods, as in the report of Gelmez et al. [24] and Zacchi et al. [25], the tocopherol contents of the extracts decreased with extraction yield increased. Additionally, ethanol is a particularly promising alternative for its higher operational safety and low toxicity, produced from renewable sources to extract high-quality oil.

In general, oxidation of lipids was a major reason for the metamorphism of edible oils, forming many kinds of secondary oxidation products. This process has an undesirable effect on the oil with the change of the chemical, sensory and nutritional properties. In this study, the induction time (IT) has been measured to evaluate the oxidation stability of oils. As shown in Table 4, the IT of the SBE-e OBO (8.22 h) was longer than the other two sample oils (7.59 h and 7.68 h). This was probably because the oils have higher levels of biological compounds such as phytosterols, tocopherols and total phenols. Moreover, the differences in the amount of UFAs (oleic acid 43.53–44.13%, linoleic acid 32.54–33.22%) may have an impact on the main oxidation products of UFAs during the process.

**Figure 2:**
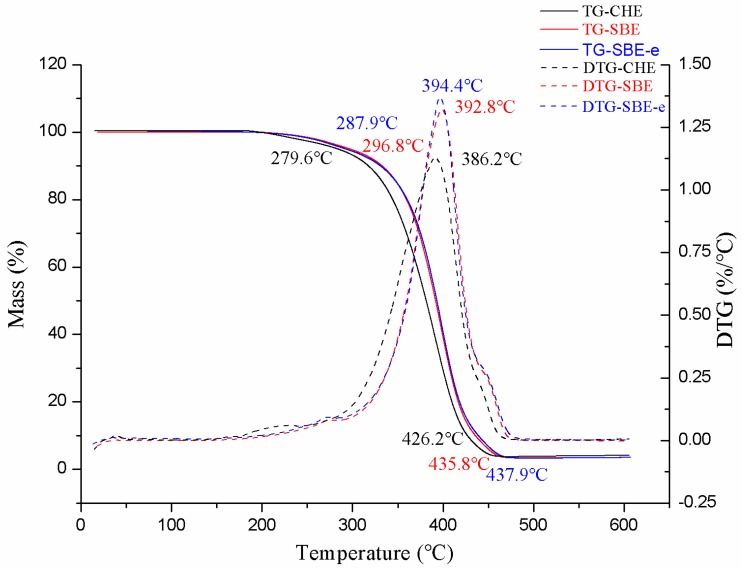
Thermal gravity(TG)/differential thermogravimetry (DTG) of OBO extracted by different methods.

Thermal gravity analysis (TGA) was also introduced to measure the thermal stability of OBO and the data are shown in Figure 2. The results indicated that OBO has a relatively stable thermodynamic properties in presence of all these samples had a 5% mass loss occurred at nearly 300 °C (279.6 °C, 287.9 °C and 296.8 °C), and a 90% mass loss for the methods of CHE, SBE and SBE-e were 426.2 °C, 435.8 °C and 437.9 °C respectively. The differential thermogravimetry (DTG) curve of OBO showed a mass-loss stage at mainly around 390 °C (386.2 °C, 392.8 °C and 394.4 °C), which could be explained by the combustion of the triglycerides accompanied by the decomposition of fatty acids at high temperature. The results also suggest that there are no significant differences in thermal stability between different extraction methods.

### 2.4. Antioxidant Capacity of the Oils

**Figure 3:**
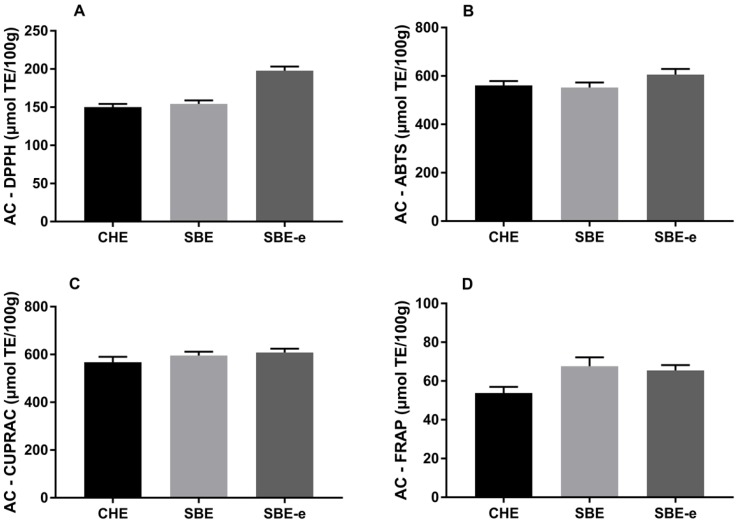
Antioxidant capacities (AC) of OBO extracted by different methods. **A**: DPPH for analyses of AC; **B**: ABTS for analyses of AC; **C**: CUPRAC for analyses of AC; **D**: FRAP for analyses of AC.

The results of DPPH, ABTS, CUPRAC and FRAP in OBO extracted by three different methods are shown in Figure 3. It is noteworthy that AC determined by four analytical procedures in OBO had a slight difference. According to Figure 3A, there were significant differences (*p* < 0.05) in the value of the CHE (150.01 ± 2.6 µM TE/100 g), SBE (154.25 ± 4.5 µM TE/100 g) and SBE-e (197.74 ± 5.6 µM TE/100 g) methods. The extraction with the cosolvent of ethanol (SBE-e) resulted in higher (*p* < 0.05) scavenging activities (DPPH) than other samples. One possible explanation for these discrepancies was the presence of phenols because of the excellent correlation (r = 0.917) with the total phenols content (TPC). Other studies have reported significantly correlation between free radical scavenging activity of vegetable oils determined by DPPH method and total tocopherol content (r = 0.70 and 0.75 for methanolic and ethyl acetate extracts) [26]. In this study, the scavenging activity of OBO samples had a similar trend as the data correlated with their tocopherol values (r = 0.915).

In ABTS (Figure 3B) tests, the differences for three methods were not as apparent as the DPPH results. In particular, for ABTS method, the obtained result of SBE-e OBO (605.99 ± 23.2 µM TE/100 g) was still higher than that of CHE OBO (560.78 ± 18.5 µM TE/100 g) and SBE OBO (552.57 ± 20.1 µM TE/100 g). Compared with DPPH assay, the correlation of samples for DPPH with their TPC values (r = 0.917) was better than that of samples for ABTS (r = 0.699). The results of CUPRAC assay for three different methods were shown in Figure 3C. There was no difference (*p* > 0.05) based on the AC results of between SBE (595.80 ± 15.6 µM TE/100 g) and SBE-e (608.09 ± 10.2 µM TE/100 g) method for CUPRAC, but significantly higher (*p* < 0.05) than CHE (567.89 ± 22.8 µM TE/100 g) method. These discrepancies between the AC results could be explained by the different mechanisms of the applied analytical methods. ABTS and CUPRAC assays are suitable to assess the AC of both lipophilic and hydrophilic antioxidants in samples, whereas DPPH assay has higher affinity toward lipophilic than hydrophilic owing to the use of a radical dissolved in organic media.

As shown in Figure 3D, there was no particular trend and difference (*p* > 0.05) on the results of SBE (67.59 ± 4.5 µM TE/100 g) and SBE-e (64.99 ± 2.7 µM TE/100 g). However, the OBO obtained by CHE method (53.74 ± 3.2 µM TE/100 g) was significantly lower (*p* < 0.05) than other two methods. In this test, the difference in FRAP from other assays was the high value of SBE method and it was attributed to the FRAP is basically a hydrophilic antioxidant assay, which does not respond to lipophilic antioxidants very well [27]. Additionally, the FRAP assay does not detect antioxidant compounds containing functional group, which is consistent with the content of bioactive compounds discussed above. The proposed analytical assays were used to determine the AC of OBO extracted by four different methods; compared with CHE method, it can be concluded that the SBE had an effect on improving AC by retaining bioactive compounds.

## 3. Material and Methods

### 3.1. Materials

Oat (*Avena sativa* L.) bran was obtained from Oats House Agriculture Development Co. (Inner Mongolia, China). Samples were roasted at 150 °C for 5 min to inactivate enzymes and ground into powders with a Wiley Mill (Thomas Scientific, Philadelphia, PA, United States). Powers passed through a 60-mesh sieve and then stored at −20 °C for further use.

Butane (≥ 99.9% purity) was purchased from Puyang Longyu Chemical Co., Ltd. (Henan, China). Other chemicals and solvents used were of either analytical or chromatographic grade, which were obtained from Fisher Scientific Chemical (Loughborough, UK), Sigma Aldrich (Steinheim, Germany) or Sinopharm Chemical Reagant Co., Ltd. (Shanghai, China).

### 3.2. Extraction Procedures

#### 3.2.1. Subcritical Butane Extraction

**Figure 4:**
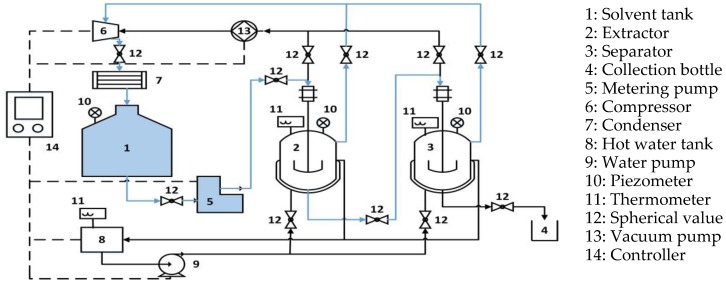
Schematic diagram of the SBE process.

Subcritical butane extraction (SBE) was performed using the apparatus (Henan Subcritical Biological Technology Co., Ltd., Anyang, China) and the schematic of this equipment is shown in Figure 4. Dried sample (500 g) was loaded into the extractor with a filter bag of 300 meshes. The air in the extractor was evacuated by a vacuum pump (13) at first, which provides an airtight and oxygen-free container for extraction. The butane was injected into the extractor (2) in the form of subcritical fluid because of a pressure difference. The liquid-to-solid ratio can be set with the assistance of a metering pump (5) and the ethanol (5% mixed solvent) was introduced through an injector as the cosolvent. The extraction was processed at a certain temperature, time, and extraction cycles (three times) under a typical pressure, they can all be controlled automatically by a controller (14). After processing, the liposoluble extract reached the separator (3) and the vaporized butane was compressed by the compressor (6) condensed to be recycled. Finally, the extracted oil was gathered at the outlet of the separator (3) and stored at 4 °C for further analysis.

#### 3.2.2. Conventional Hexane Extraction

Conventional hexane extraction (CHE) was performed by means of an overhead stirrer (IKA, 200 digital) and a heating bath to simulate the production of edible oil in industry. Sample was extracted with hexane in a flask at 55 °C for 2 h and the mixture was centrifuged at 8000 rpm for 20 min. After extraction, the mixture of solvent and extract was separated in a rotary evaporator (BUCHI, R-100) and the solvent was evaporated under a stream of N_2_.

The extraction yield was determined gravimetrically by the mass of extracted oil divided by the mass of dry oat bran taken in the extraction, namely Equation (2):

(2) $$\mathrm{The}\;\mathrm{extraction}\;\mathrm{yield}\;\left(\mathrm{wt}\;\%\right)=\frac{\mathrm{extracted}\;\mathrm{oil}\;\left(g\right)}{\mathrm{dried}\;\mathrm{material}\;\left(g\right)}\;\times 100$$

### 3.3. Response Surface Methodology Design

Based on preliminary single-factor experiments, ranges of liquid-to-solid ratio, extraction time, extraction temperature and the number of extraction cycles were determined. Box-Behnken design (software Design-Expert 10, Stat-Ease, Inc., Minneapolis, MN, United States) was selected for the study because it requires fewer run compared to central composite design (CCD) in case of three variables. Based on single-factor experiments, three key independent variables were determined to be liquid-to-solid ratio (*X*_1_), extraction time (min, *X*_2_) and extraction temperature (°C, *X*_3_). The extraction yield (*Y*) was taken as the response values in the optimization experiments. Five replicates at the center points to evaluate the pure error. Regression analysis was performed for the experimental data and explained by the following second-order polynomial regression Equation (3):

(3) $$Y=\beta_{0}+\overset{3}{\underset{i=1}{\sum }}\beta_{i}X_{i}+\overset{3}{\underset{i=1}{\sum }}\beta_{ii}X_{i}^{2}+\overset{2}{\underset{i=1}{\sum }}\overset{3}{\underset{j=i+1}{\sum }}\beta_{ij}X_{i}X_{j}$$

Here *Y* presents the response values, and *β*_0_, *β_i_*, *β_ii_*, and *β_ij_* are the regression coefficients of variables for constant, linear, quadratic, and interaction terms, respectively. *X_i_* and *X_j_* are independent variables (*i* ≠ *j*). *X_i_*^2^ and *X_i_X_j_* are the quadratic and interaction terms, respectively. The fitness of the polynomial model equation is expressed by the coefficient of determination *R*^2^, the statistical significance is confirmed by *F*-test at a probability (*P*) of 0.001, 0.01, or 0.05.

### 3.4. Characterization of Oat Bran Oil

The standards of ISO (International Organization for Standardization) were used for the determination of acid value (ISO 660, 2009), iodine value (ISO 3961, 2013), peroxide value (ISO 3960, 2007). The oxidative stability was determined by Rancimat (Metrohm 743, Herisan, Switzerland) according to ISO 6886, 2006. The oil samples (3 g) were placed in the Rancimat apparatus at a temperature of 120 °C, under a constant air flow of 20 L·h^−1^. The induction time (IT) was recorded automatically by apparatus software.

Oryzanol content in the sample was determined spectrophotometrically (V-630 Spectrophotometer, JASCO, Tokyo, Japan) according to the method described by Zullaikah et al. [21]. The sample was dissolved in trichloromethane and diluted with ethanol, the absorbance was measured at 327 nm wavelength.

Squalene content was analyzed with the Agilent 7890B GC (Agilent Technologies, Inc., Palo Alto, CA, United States) equipped with a FID detector and a capillary column of HP-5. The detector and injection temperature were 300 °C. The initial oven temperature was 130 °C and then increased to 230 °C with a rate of 20 °C/min. The final oven temperature was 270 °C elevated by a ramp rate of 3 °C. Helium (99.99%) was used as the carrier gas and high purity nitrogen was used as the make-up gas. An external standard method was used to measure the concentration of squalene quantitatively.

Phytosterols were analyzed with an Agilent 7890A GC system (Agilent Technologies, Inc., Palo Alto, CA, USA) equipped with a flame ionization detector and an HP-5 capillary column. Oil samples (0.2 g) were refluxed with 2.0 M KOH (10 mL) in ethanol to saponification. The mixture was placed in drying oven at 60 °C for 1h and stirred every 15 min. Hexane was used to extract the unsaponifiable matter twice. The hexane phase collected by centrifugation and washed with deionized water. The solvent was dried with anhydrous sodium sulfate and 5 mL was taken to remove the hexane under the help of nitrogen. Then 100 µL anhydrous pyridine and 100 µL BSTFA + TMCS (99 + 1) were added and heated at 105 °C for 20 min for derivatization. Additional, the oven temperature was held at 190 °C for 2 min and ramped to 230 °C at the rate of 20 °C/min for 5 min. Then it ramped a second time to 255 °C at the rate of 40 °C/min and held for 25 min. Helium was used as the carrier gas at a flow rate of 1.0 mL/min. The temperature of injection and detector were both 300 °C. 

Tocopherol content of the oil samples was determined by a high-performance liquid chromatography (HPLC) method. Samples (5.0 mg) were dissolved in hexane and filtered through a 0.45 µm membrane filter. The samples were analyzed with a LC-20A HPLC (Shimadzu Crop., Kyoto, Japan) equipped with an ultraviolet detector. The mobile phase was hexane:isopropyl alcohol (98:2, *v*/*v*). The liquid chromatography conditions were: run time, 25 min; column temperature, 27 °C; flow rate, 0.9 mL/min; absorption wavelength, 292 nm. Peaks were quantified by area compared to a mixture of standards.

Total phenols content (TPC) were determined according to the method described by Szydłowska et al. [28]. Oil samples (5.00 g) were dissolved in hexane (15 mL) and extracted with methanol (5 mL) for 3 times. Wash the methanolic extracts with hexane (25 mL) and take 1 mL of it to 10 mL calibration flask. 0.5 mL of Folin-Ciocalteu reagent and 1 mL saturated sodium carbonate solution were added, shaking for 3 min, and made up with redistilled water. Keep solutions in the dark for 1 h and centrifuged at 8000 rpm for 10 min, the absorbance was measured at 760 nm wavelength against a reagent blank. Calibration curves were prepared for working solutions of gallic acid in the concentration range of 1–10 µg/mL. Five calibration curves were plotted using the least-squares method resulting in equation *y* = (0.095 ± 0.005)*x* + (0.033 ± 0.004), *R*^2^ = 0.9991, R.S.D._slope_ = 0.9% (*n* = 5).

### 3.5. Determination of Fatty Acid

Oil samples were derivatized into fatty acid methyl esters and analyzed by gas chromatography (GC). Samples (0.3 g) were dissolved in hexane and esterified with KOH ethanol solution, stirred, held in drying oven at 60 °C for 30 min. The analysis was carried out on an Agilent 7890A GC system (Agilent Co., Palo Alto, CA, USA) equipped with FID and a capillary column. Qualitative analysis was performed according to the retention time of fatty acid curves compared to standard methyl esters (Sigma Aldrich Co., Steinheim, Germany). Quantitative analysis of fatty acids was determined by measuring peak area.

### 3.6. Antioxidant Capacity Determination

The antioxidant capacity (AC) of the OBO was evaluated by means of four analytical methods: DPPH, ABTS, CUPRAC, FRAP, originally developed by Brand et al. [29], Re et al. [30], Apak et al. [31], Benzie and Strain [32], respectively. The spectra of the solutions were measured by Unico UV-2800 (Shanghai, China).

Calibration curves were prepared using working solutions of Trolox in methanol of concentrations 0.020–0.100, 0.010–0.050, 0.003–0.015 and 0.005–0.040 µmol/mL for DPPH, ABTS, CUPRAC and FRAP methods, respectively. Five calibration curves were plotted on the same day. The least-squares method was applied to calculate the following lines: *y* = (580.15 ± 2.60)*x* + (4.20 ± 0.50) for DPPH method, *y* = (251.95 ± 1.24)*x* + (2.66 ± 0.28) for ABTS method, *y* = (0.179 ± 0.015)*x* + (0.118 ± 0.009) for CUPRAC, and *y* = (45.63 ± 0.25)*x* + (0.028 ± 0.004) for FRAP. The calculated coefficients were 0.9993, 0.9959, 0.9976 and 0.9987, with the relative standard deviations (*n* = 5) of the slopes were 1.06%, 1.53%, 0.82% and 0.97%, respectively. 

The extracts of the OBO were obtained in methanol for determination of antioxidant capacity. Add methanol (5 mL) into oil samples (3.00 g) in test tubes and stirred for 30 min at 20 °C in the dark with a shaker (85BS, Zhichu Instrument Co., Ltd., Shanghai, China). Then take the extracts from oils with centrifugation and stored in refrigerator for AC analysis.

### 3.7. Statistical Analyses

All experiments were performed in triplicate and all the results were expressed as mean value ± SD (standard deviation). An analysis of variance was performed using SPSS Statistics software version 17.0 (SPSS, Chicago, IL, USA). Data collected from the SB were carried out using the software Design-Expert 10.0.4 (Stat-Ease, Inc., Minneapolis, MN, USA) and Origin 2017 (OriginLab, Northampton, MA, USA).

## 4. Conclusions

Subcritical butane extraction (SBE) technology was firstly applied to the extraction of OBO from oat bran, and the optimization of SBE was performed with a statistical method based on the RSM. The optimum SBE condition was as follows: liquid-to-solid ratio 4.30, extraction time 48.15 min, and extraction temperature 46.52 °C. The OBO was rich in unsaturated fatty acids (mainly oleic acid and linoleic acid). There were negligible differences in fatty acid composition and physicochemical characteristics compared to CHE method. Furthermore, in this study, a promising alternative SBE-e was developed to extract OBO as an improvement of SBE. It was found that the cosolvent (ethanol) had a positive effect on the yield, bioactive compounds (phytosterols and phenols) and antioxidant capacity (AC) in the extracted OBO. Overall, OBO showed desirable physicochemical characteristics, thermal stability, fatty acids composition, bioactive compounds, and AC; it may be an acceptable substitute for edible oil and SBE method appeared to be preferably suitable for OBO extraction.

## Figures and Tables

**Table 1:** Box-Behnken design (BBD) matrix and the response values of the extraction yield of OBO.

RUN	Independent Variable			Yield (%)	
	*X*_1_ (Liquid-to-solid ratio)	*X*_2_ (Time, min)	*X*_3_ (Temperature, °C)	Experimental	Predicted
1	4	60	60	5.86 ± 0.23	5.69
2	5	40	20	5.23 ± 0.17	5.08
3	3	20	40	5.13 ± 0.21	5.02
4	4	60	20	4.96 ± 0.11	5.00
5	4	40	40	6.04 ± 0.28	5.92
6	3	40	60	5.48 ± 0.18	5.63
7	3	40	20	4.54 ± 0.09	4.48
8	5	20	40	5.25 ± 0.32	5.22
9	5	60	40	5.76 ± 0.24	5.87
10	4	40	40	5.91 ± 0.30	5.92
11	3	60	40	5.46 ± 0.12	5.49
12	4	40	40	5.88 ± 0.33	5.92
13	4	20	20	4.12 ± 0.13	4.29
14	5	40	60	5.55 ± 0.29	5.61
15	4	40	40	5.99 ± 0.14	5.92
16	4	40	40	5.77 ± 0.26	5.92
17	4	20	60	5.32 ± 0.22	5.28
CHE	4	120	55	5.75 ± 0.26	

**Table 2:** The analysis of variance (ANOVA) for the quadratic polynomial mode.

Source ^1^	Coefficient Estimate	Standard Error	Sum of Squares	Df	Mean Square	*F*-Value	*p*-Value ^2^
Model			4.27	9	0.47	18.18	0.0005
Intercept	5.92	0.072		1			
*X*_1_	0.15	0.057	0.17	1	0.17	6.67	0.0363
*X*_2_	0.28	0.057	0.62	1	0.62	23.62	0.0018
*X*_3_	0.42	0.057	1.41	1	1.41	54.10	0.0002
*X*_1_ *X*_2_	0.045	0.081	0.0081	1	0.0081	0.31	0.5947
*X*_1_ *X*_3_	−0.16	0.081	0.096	1	0.096	3.68	0.0964
*X*_2_ *X*_3_	−0.075	0.081	0.022	1	0.022	0.86	0.3839
*X*_1_^2^	−0.19	0.079	0.15	1	0.15	5.92	0.0452
*X*_2_^2^	−0.33	0.079	0.45	1	0.45	17.21	0.0043
*X*_3_^2^	−0.53	0.079	1.17	1	1.17	44.75	0.0003
Residual			0.18	7	0.026		
Lack of Fit			0.14	3	0.046	4.27	0.0975
Pure Error			0.043	4	0.011		
SD	0.16		*R* ^2^	0.9590			
Mean	5.43		Adj. *R*^2^	0.9062			
CV (%)	2.98		Pred. *R*^2^	0.4845			
PRESS	2.29		Adeq. Precision	13.123			

Df, degree of freedom; SD, standard deviation; CV, coefficient of variation. ^1^ *X*_1_, liquid-to-solid ratio; *X*_2_, extraction time; *X*_3_, extraction temperature. ^2^ *p* < 0.01 indicates high statistical significance; *p* < 0.05 indicates statistical significance; *p* > 0.05 indicates statistical non-significance.

**Table 3:** Oil yield and fatty acid composition of OBO extracted by different methods.

Extraction Method	Yield (%)	C16:0	C18:0	C18:1	C18:2	C18:3	Others	UFA
SBE	6.05 ^ab^ ± 0.15	16.03 ^a^ ± 0.03	1.89 ^a^ ± 0.04	44.09 ^ab^ ± 0.18	32.74 ^b^ ± 0.11	0.86 ^a^ ± 0.07	4.39 ^b^ ± 0.12	77.61 ^a^
SBE-e	6.42 ^a^ ± 0.23	15.87 ^a^ ± 0.12	1.68 ^b^ ± 0.10	43.53 ^c^ ± 0.11	33.22 ^a^ ± 0.09	0.71 ^ab^ ± 0.06	4.99 ^a^ ± 0.09	76.87 ^a^
CHE	5.75 ^b^ ± 0.26	15.91 ^a^ ± 0.04	1.86 ^a^ ± 0.04	43.84 ^b^ ± 0.19	32.68 ^b^ ± 0.12	0.63 ^b^ ± 0.15	5.08 ^a^ ± 0.19	77.80 ^a^

Values were reported as the mean ± standard deviation based on triplicate analyses. Means followed by the same letter indicate no significant difference (*p* > 0.05). SBE, subcritical butane extraction; SBE-e, subcritical butane extraction with the cosolvent of ethanol; CHE, conventional hexane extraction; UFA, unsaturated fatty acid.

**Table 4:** Physicochemical characteristics of OBO extracted by different methods.

	Methods	SBE	SBE-e	CHE
Properties				
Acid value (mg/g oil)		7.16 ^a^ ± 0.21	7.45 ^a^ ± 0.23	7.26 ^a^ ± 0.26
Peroxide value (meq. O_2_/kg oil)		0.74 ^a^ ± 0.04	0.81 ^a^ ± 0.10	0.68 ^a^ ± 0.04
Iodine value (g/100 g oil)		102.78 ^a^ ± 0.53	102.83 ^a^ ± 0.72	103.63 ^a^ ± 0.56
Induction time (h, 120 °C)		7.68 ^b^ ± 0.08	8.22 ^a^ ± 0.15	7.59 ^b^ ± 0.13
Oryzanol (mg/g)		4.28 ^a^ ± 0.23	4.16 ^a^ ± 0.19	4.40 ^a^ ± 0.17
Squalene (mg/kg)		43.40 ^a^ ± 0.09	43.53 ^a^ ± 0.15	43.47 ^a^ ± 0.12
Phytosterols (mg/g)		2.47 ^b^ ± 0.10	2.99 ^a^ ± 0.14	2.55 ^b^ ± 0.14
Tocopherol (mg/100 g)		15.91 ^b^ ± 0.32	18.69 ^a^ ± 0.16	15.77 ^b^ ± 0.28
Total phenols content (GA mg/100 g)		6.84 ^b^ ± 0.32	9.63 ^a^ ± 0.26	6.47 ^b^ ± 0.24

Values were reported as the mean ± standard deviation based on triplicate analyses. Means followed by the same letter indicate no significant difference (*p* > 0.05). SBE, subcritical butane extraction; SBE-e, subcritical butane extraction with the cosolvent of ethanol; CHE, conventional hexane extraction.
